# Evaluations of knowledge, skills and practices of insulin storage and injection handling techniques of diabetic patients in Ethiopian primary hospitals

**DOI:** 10.1186/s12889-020-09622-4

**Published:** 2020-10-12

**Authors:** Adeladlew Kassie Netere, Eyayaw Ashete, Eyob Alemayehu Gebreyohannes, Sewunet Admasu Belachew

**Affiliations:** Department of Clinical Pharmacy, School of Pharmacy, College of Medicine and Health Sciences, University of Gondar, Lideta Sub City Kebele 16, Gondar, Ethiopia

**Keywords:** Insulin, Injection, Knowledge, Practice, Storage, Primary hospitals, Ethiopia

## Abstract

**Background:**

Insulin is an effective therapeutic agent in the management of diabetes, but also sensitive to the external environment. Consequently, diabetic patients’ adherence to insulin delivery recommendations is critical for better effectiveness. Patients’ lack of knowledge, skill and irrational practices towards appropriate insulin delivery techniques may end up in therapeutic failure and increase costs of therapy. The aim of this study was to evaluate patients’ knowledge, skills and practices of insulin storage and injection techniques.

**Methods:**

An interview-based cross-sectional study was conducted through purposive selection of participants in Northwest Ethiopian primary hospitals from March 1 to May 30, 2019. Levels of knowledge were assessed with right or wrong responses, while practice was measured by using a 4-point Likert scale structured questionnaire collected via face-to-face interviews. Likewise, a five-point observational (demonstration) techniques checklist employed to assess patients’ skills.

**Results:**

Among 194 patients approached, 166 participants completed the survey giving a response rate of 85.6%. More than half of the respondents (54.8%) were males and the mean age (±SD) was 38.5 ± 13.8 years. The overall patients’ median knowledge and practice levels on insulin storage and handling techniques were moderately adequate (64.3%) and fair (55.4%), respectively. In patients’ skill assessments, 94.6% correctly showed injection sites, 70% indicated injection site rotations, and 60.75% practiced injection site rotations. Education (*P < 0.001*), duration of insulin therapy (*P* = 0.008), and duration of diabetes (*P* = 0.014) had significant impact on knowledge level. Education (*P* < 0.001), occupation (*P* < 0.001), duration of insulin therapy (*P* = 0.001), duration of diabetes (*P* = 0.036) and patients’ knowledge level (*P* < 0.001) were found to have a significant effects on the patients’ practice levels. A Mann-Whitney U test also disclosed that residency, ways to get insulin and mocked injection technique during the first training had significant effects on patients’ knowledge levels.

**Conclusion:**

The current study revealed that patients had moderately adequate knowledge and fair practice levels on insulin storage and handling techniques. However, patients missed important insulin administration skills. This study highlights the need of regular public health education so as to enhance the patients’ knowledge, skill and practice levels on insulin handling techniques.

## Background

Insulin is an effective drug for the control of blood sugar level. It is the mainstay treatment for patients with type 1 diabetes (T1DM) and it is often used as an adjuvant to oral hypoglycemic agents in patients with type 2 diabetes (T2DM) who failed to achieve target blood glucose levels [1, 2]. Despite its effectiveness, insulin is a very sensitive drug and can be affected by many external factors. For instance, if left in unconducive environment it can be easily destroyed and lose its efficacy [3–5]. To handle this liability and get the ultimate benefits from insulin, implementing the correct insulin delivery recommendations is crucial. Such recommendations can be found as an important instrument in mitigating the mal practices and ensures the safe insulin handling and delivery techniques for DM patients’ [1].

The primary goal of diabetes management is to achieve the blood sugar level within the target ranges. In an effort to meet this target, an appropriate delivery of insulin is essential [1]. Diabetic patients are very likely to benefit from good adherence and proper implementation of specific recommendations. One such recommendation is the Forum for Injection Technique and Therapy: Expert Recommendations (FITTER) [6] workshop held in Rome, Italy in 2015. The FITTER help to enhance therapeutic outcomes and lower costs of therapy [1].

Disinterest with or ignorance of the guidelines for insulin handling and delivery techniques could be a result of poor knowledge, skill and practice behavior. This, in turn, could facilitates delayed drug action (lack of insulin stability and potency), therapeutic failure and increased healthcare expenditures [3, 5, 7–9]. Evidence-based skills and good practices of insulin handling techniques are warranted for better treatment outcomes. This is especially true in resource limited settings; such patients rely on scientific underpin recommendations to a little extent, if at all, and depend on behavior and customs as evidence [2]. In low income communities such as those in the Eastern African region; in addition, to limited access to the updated guidelines and recommendations [10], translating the guidelines and recommendations to local languages for better patient utilization is challenging. Moreover, unaffordability and inaccessibility of important storage equipment coupled with the unfavorable weather conditions in this region could further compromise the overall quality of insulin.

Furthermore, negative attitudes of diabetic patients towards insulin administration may further compromise their interest to look for appropriate instructions of storage and handling techniques. Thus, educating and changing their perceptions, beliefs and attitudes towards storage and administering techniques needs to be part of an intervention, which aimed to improve treatment outcomes in diabetes patients [11]. In addition to implementing the recommendations, healthcare professionals (HCP) could also play an important role for counseling patients on the best injection techniques prior to the start of injection therapy [12–14]. Demonstration or education, which have a bi-directional nature leads to better up-take of instructions by patients [15, 16]; and any decision needs to be mutually agreed between patients and the HCPs [12, 17]. In addition to improving therapeutic outcomes, regular training and assessments of practices of insulin handlings, storages and injections can also prevent injection site discomforts [18].

To the best of the authors’ knowledge and a literature search, studies that evaluated the knowledge, skills and practices of insulin storage and injection technique of patients in the study area are lacking. With this, the purpose of the current study was to evaluate the knowledge, skills and practices of insulin storage and injection technique of patients in the primary hospitals of Northwest Ethiopia.

## Methods

### Study design and setting

An institutional-based cross-sectional study was conducted in randomly selected primary hospitals in Northwest Ethiopia, located Northwest of Addis Ababa. Structured questionnaire-based interviews were administered from March 1 to May 30, 2019. The study area had one comprehensive specialized referral and teaching hospital, one private general hospital, ten primary hospitals, and a number of health centers. The study participants were recruited from the selected primary hospitals located in the towns of Addis Zemen, Debark, Wogera, Kolladiba, Chilga and Metema.

### Source population, sample size determination and sampling procedure

The source population was all diabetic patients who had been treated with insulin in the study area. The study population included those diabetic patients who were using insulin as their primary therapy or as additional therapy and visited those hospitals during the study period. Patients or patient caregivers who were 18 years of age and above were included in the study. Patients had to be on insulin treatment for at least 1 month and refilling insulin prescriptions at one of the hospitals. Patients who did not consent to participate in the study or those who were seriously ill, unable to hear or speak, physically disabled, having dementia or cognitive impairment and difficulty of getting consent were excluded from the study. A convenient sampling technique was used to collect data.

### Data collection instruments, procedure and management

The data collection format was initially prepared in English. It was then translated to the local language (Amharic) and back-translated to English to ensure proper meaning. Trained pharmacy professionals, under investigators’ daily supervision collected the data. The questionnaire focused on socio-demographic and related information; experiences, practices and knowledge of insulin storage and handling techniques; and an observational checklist of patients’ skills related to insulin self-administration (supplementary material 1). Both the practice and the knowledge questions contained 14 items. Practices were measured by Likert scale type (Never = 1; Sometimes = 2; Often/Usually =3; Always = 4) and graded as poor, fair and good for scores of < 50% (< 28), 51–75% (29–42) and > 75% (> 42) out of 56 points, respectively. The knowledge of respondents’ was measured with dichotomous outcomes as “right” in participants who answered a question correctly and “wrong” in those who answered a question incorrectly. Finally, all the responses were summed up to an overall score and categorized into three levels. Scores of > 75% (> 10.5), 51–75% (8–10.5), and < 50% (< 7) out of 14 points were said to be adequate, moderately adequate and inadequate, respectively [19]. The respondents’ skills were measured through a checklists of five observational (demonstration) techniques related to insulin self-administration procedures. Based on American diabetic association’s standards of care and publicly available information on insulin administration [20, 21], the checklist was marked as correct, incorrect, and skipped and given scores of 2, 1, and 0, respectively. If a patient or a caregiver correctly performed all the critical steps, the observation (demonstration) was considered as correct; if any of the critical step was missed or performed incorrectly, the demonstration was considered as incorrect; and it is considered as skipped if any of the steps was jumped.

### Data entry, analysis, and interpretation

Data was entered and analyzed using statistical package for social sciences (IBM-SPSS), version 22.0 [22]. Frequency, percentages and median were used to describe the variables in univariate analysis, while Chi-square, Mann-Whitney U and Kruskal-Wallis H tests were used to describe and test the statistical significance of variables in bivariate analyses. Pairwise multiple comparisons were done for those groups who had significant knowledge and practice median differences on the Kruskal-Wallis H test. The knowledge and practice data were transformed into categorical values. The knowledge levels were classified as inadequate, moderately adequate and adequate knowledge; and practice levels were expressed as poor, fair and good practices. A *p*-value of < 0.05 and a 95% confidence interval (CI) were used as cut-off points for determining the statistical significance of associations among different variables. Spearman’s correlation coefficient test was done to assess the degree of correlation between the patients’ knowledge, and their practice levels in their insulin handling techniques and injection practices.

### Data quality control

Before the commencement of data collection, a pretest was done on 15 patients from one of the randomly selected primary hospitals. These patients were not included in the final data analysis. Important amendments were made and modified based on the pre-test findings. The data accuracy and completeness were consistently checked by using double entry, and errors and omissions were corrected.

### Informed consent and confidentiality

Before data collection had begun, the aims of the study were clearly explained to participants. In addition, a written informed consent was obtained from each of the included participants. The participation in the study was completely volunteer and they were free to withdraw from the study any stage without forwarding any justification. The respondents were interviewed and observed keeping their privacy. All information was kept confidential with no participant identifiers.

## Result

### Respondent characteristics

A total of 194 patients were approached, of which 28 did not give consent to take part. Thus, the remaining 166 participants agreed and completed the survey giving a response rate of 85.6%. The number of male participant showed preponderance (54.8%). Most of the respondents (29.5%) were between the ages of 18–27 years with a mean age of 38.5 ± 13.8 years. More than half of the participants (56.6%) were married; and about 53% had attended their primary (1–8 grades) education and above*.* Nearly half (48.8%) of the participants claimed as they have lived with DM for about 1–5 years with a mean (±SD) of 2.5 ± 0.9 years. Marked number of (58.4%) of respondents used insulin therapy for about 1–5 years with a mean (±SD) duration of 2.3 ± 0.8 years. More than half (55.4%) of the participants obtain insulin merely through purchase (Table 1).

**Table 1:** Distribution of selected characteristics of patients with diabetes mellitus by sex (*N* = 166); 2019

Variables	Total	Male	Female	*P*-value
	N (166) %(100.0)	N (91) % (54.8)	N (75) %(45.2)	
Age in years				
18–27	49 (29.5)	28	21	0.778
28–40	43 (25.9)	22	21	
41–50	43 (25.9)	22	21	
> 50	31 (18.7)	19	12	
Mean ± SD	38.5 ± 13.8			
Residence				
Rural	86 (51.8)	43	43	//
Urban	80 (48.2)	48	32	
Marital status				
Single	56 (33.7)	31	25	**0.02**
Married	94 (56.6)	52	42	
Divorced	10 (6)	8	2	
Widowed	6 (3.6)	0	6	
Occupation				
Farmer	34 (20.5)	31	3	**< 0.001**
Employer	31 (18.7)	18	13	
Merchant	38 (22.8)	21	17	
Housewife	36 (21.7)	8	28	
Student	27 (16.3)	13	14	
Educational status				
Illiterate	48 (28.9)	27	21	0.546
Read & write only	30 (18.1)	17	13	
Primary and secondary education	52 (31.3)	31	21	
College & above	36 (21.7)	16	20	
Duration of DM (in years)				
0.25–1	15 (9)	5	10	**0.002**
> 1–5	81 (48.8)	38	43	
> 5–10	40 (24.1)	23	17	
> 10	30 (18.1)	25	5	
Mean ± SD	2.5 ± 0.9			
Duration of Insulin therapy (in years)				
0.25–1	16 (9.6)	6	10	**< 0.001**
> 1–5	97 (58.4)	44	53	
> 5–10	39 (23.5)	27	12	
> 10	14 (8.4)	14	0	
Mean ± SD	2.3 ± 0.8			
Getting of insulin				
Freely	74 (44.6)	39	35	//
Payment	92 (55.4)	52	40	

### Knowledge score

**Fig. 1:**
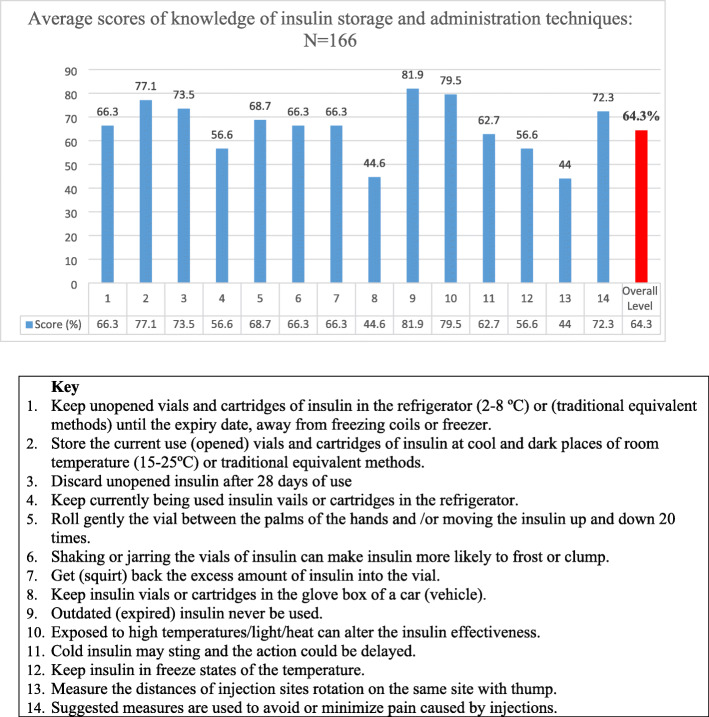
Knowledge of insulin storage and administration techniques

The overall median (IQR) knowledge level of the study subjects about insulin storage and handling techniques was 9 (7.8–11), out of 14 (64.3%). Most of the participants (44%) had moderately adequate knowledge, while nearly one-third of them (31.3%) had adequate knowledge, and the rest of the patients (24.7%) had inadequate knowledge levels. Large number of patients (82%) knew that outdated (expired) insulin should not be used. Less than half (44%) of the respondents were aware of the acceptable distance in regards to injection site rotation on the same site, which is known to be one thumb (Fig. 1).

A non-parametric Kruskal Wallis H test was performed to compare the effect of educational status, duration of insulin therapy, and duration of diabetes on patients’ level of knowledge for insulin storage and handling techniques. The test showed that the levels of education X^**2**^(3) =18.9, *P* < 0.001; duration of insulin therapy X^**2**^(3) =11.7, *P* = 0.008; and duration of diabetes X^**2**^(3) =10.7, *P* = 0.014 had significantly effects on patients’ knowledge level. The Pairwise multiple comparisons at an adjusted alpha level of 0.0125 were used to compare all pairs of groups, and patients who achieved colleges and above (Median (Mdn) =12) had a higher median knowledge level than illiterates (Mdn = 8), *P* < 0.001. Patients who had been on insulin for 5–10 years (Mdn = 10) had a better knowledge level as compared with who had been on for 3 months to 1 year (Mdn = 8), *P* = 0.001 (Table 2).

**Table 2:** Kruskal Wallis H test for predictor variables on the level of knowledge and practices on insulin storage and injection differences among respondents’ in northwest Ethiopia primary hospitals, Gondar, 2019 (*N* = 166)

Variables	Knowledge score			Practice score		
	Median (IQR)	Test Statistics (X^**2**^), (df)	*P*-value	Median (IQR)	Test Statistics (X^**2**^), (df)	*P*-value
Educational status						
Illiterate	8 (6–10)	18.89, (3)	< 0.001**	28 (27–30)	25.86, (3)	< 0.001**
Read & write only	9 (8–11)			28 (24–33)		
**1**^**ry**^ and **2**^**ndry**^ education	9.5 (8–10)			31 (29.3–35)		
College & above	12 (8–13)			32 (29.3–34)		
Occupation						
Farmer			0.076	28 (27–31.3)	23.24, (4)	< 0.001**
Employer				32 (28–35)		
Merchant				29 (25.8–31)		
Housewife				31 (28–34)		
Student				32 (31–34)		
Years of insulin therapy						
0.25–1	8 (6–9)		0.008**	28.5 (21.3–30.8)	15.85, (3)	0.001*
> 1–5	9 (7–10.5)	11.71, (3)		31 (28.5–34)		
> 5–10	10 (8–12)			29 (27–31)		
> 10	11 (8.5–12)			28.5 (27–32.5)		
Years of disease						
0.25–1	8 (6–10)	10.67, (3)	0.014*	30 (22–32)	8.55, (3)	0.036*
> 1–5	9 (7–10)			31 (28–34)		
> 5–10	10 (8–12)			30.5 (27.3–33.8)		
> 10	10.5 (7.8–12)			28 (27–32.5)		
Knowledge level						
Adequate (> 10.5)	**//**	**//**	**//**	29 (23–31)	19.26, (2)	< 0.001**
Moderate (8–10.5)				30 (28–32.5)		
Inadequate (< 7)				32.5 (29–35)		

* Statistically significant effect on patients’ knowledge and practice scores at ***p*** **= 0.05**

**With Pairwise multiple comparisons of Kruskal Wallis 1-way ANOVA (k-samples) there is a significant difference among the groups

To determine the difference in knowledge level the differences of the patients’ overall knowledge levels between the two group predictor variables such as residency, ways to get insulin, and mocked injection technique during first training, we employed a Mann-Whitney U test. Significant difference in the patients’ overall knowledge level across different in residency (*P* = 0.001), ways to get insulin (*P* < 0.001) and mocked injection technique during first training (*P* = 0.016) was noted. The median knowledge score level of urban dwellers (Mdn = 10) was higher than those of rural dwellers (Mdn = 8). Similarly, patients charged for insulin (Mdn = 10) had a better knowledge level than those who obtain insulin for free (Mdn = 8). Patients who mocked injection technique during first training (Mdn = 9.5) had slightly better scores compared with those who did not (Mdn = 8.5) (Table 3).

**Table 3:** The Mann-Whitney U test for the median scores of level of knowledge differences of between categories of predictor variables (*N* = 166)

Variables	Overall knowledge score			
	Median (IQR)	Mann-Whitney U test	Z-score	*P*-value
Sex				
Male				0.291
Female				
Residence				
Urban	10 (8–12)	2410	−3.36	0.001*
Rural	8 (7–10)			
Getting of insulin				
Free	8 (7–10)	1874	−5.01	< 0.001*
Payment	10 (9–12)			
Trained on insulin injection				
Yes				0.075
No				
Mocking the injection technique during first training				
Yes	9.5 (8–12)	1959.5	−2.41	0.016*
No	8.5 (6–10)			

* Statistically significant effect on patients’ knowledge levels at ***p*** **< 0.05**

### Practice score

**Fig. 2:**
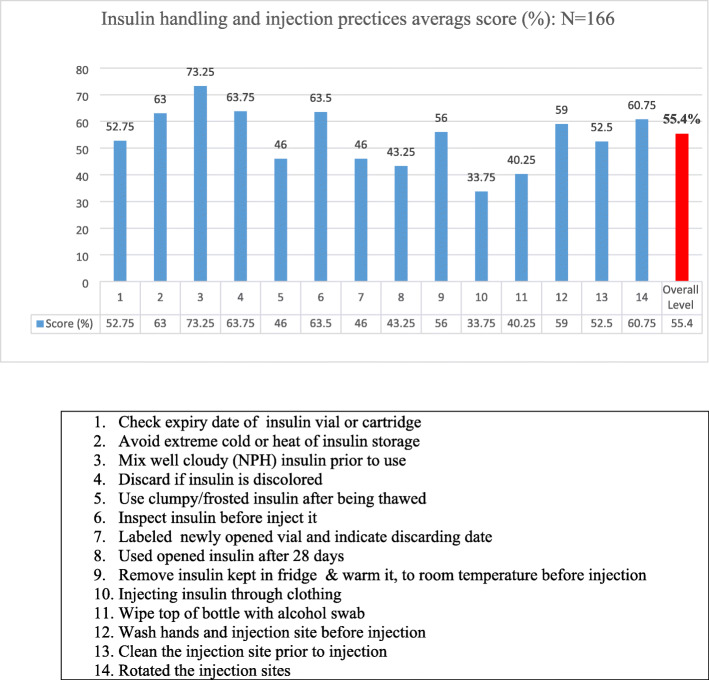
Insulin handling and injection experiences and practices

The participants’ insulin storage and injection practices were assessed by using the stated 14-item questionnaire. The median (IQR) practice level of study subjects was 31 (28–33.3) out of 56 (55.4%). Most of the participants (64.5%) had fair practice. Only 1.2% of them had good practice and the rest (34.3%) portrayed poor practice. As illustrated in Fig. 2, the majority of the patients (73.3%) mixed the cloudy insulin Neutral Protamine Hagedorn (NPH) prior to use. One-third (33.8%) of patients had ever injected their insulin through their clothes.

A Kruskal-Wallis H test identified the effects of different potential predictor variables on patients’ level of practice for insulin storage and handling techniques. Education X^**2**^(3) =25.9, *P* < 0.001; occupation X^**2**^(4) =23.2, *P* < 0.001; duration of insulin therapy X^**2**^(3) =15.9, *P* = 0.001; duration of diabetes X^**2**^(3) =8.6, *P* = 0.036; and patients knowledge levels X^**2**^(2) =19.3, *P* < 0.001 were significantly alter the patients’ practice levels. As to the Pairwise multiple comparisons at an adjusted alpha level of 0.0125 for education levels, patients who completed primary and secondary educations (Mdn = 31) had better practice levels than those who were not educated (Mdn = 28), *P* = 0.001. Those who achieved colleges and above (Mdn = 32) exhibited higher practice levels than those who did not educated (*P* < 0.001), and those who read and write only (Mdn = 28), *P* = 0.01. The Pairwise multiple comparisons at an adjusted alpha level of 0.01 for occupations indicated that students (Mdn = 32), compared with farmers (Mdn = 28), *P* = 0.003 and merchants (Mdn = 29), *P* = 0.002, correctly demonstrate insulin handling techniques. The pairwise multiple comparisons at an adjusted alpha level of 0.017; patients who scored adequate knowledge level (Mdn = 32.5) had better practice levels compared with those having moderate adequate (Mdn = 30), *P* = 0.008 and inadequate knowledge levels (Mdn = 29), *P* < 0.001 (Table 2). A Spearman’s correlation was run to determine the relationship between the patients’ knowledge level in their insulin handling techniques and their practice of insulin injections. The test showed as there was a moderate, positive correlation between the knowledge and practice level (*r*_*s*_ *= 0.425*, *P* < 0.001).

### Insulin self-administration skill assessment

Based on the observational checklists used to assess the patients’ skills related to insulin self-administration, a large numbers of participants (94.6%) correctly demonstrated the injection sites and about 70% of the participants properly indicated the pattern of injection site rotations. In contrast, about half of the respondents either performed incorrectly or skipped very critical and important steps such as shaking of cloudy NPH insulin, skin pinching and 45^**o**^ injection skill, and drawing of insulin from the vials (Table 4).

**Table 4:** Observational checklist of patients’ skill related to self-insulin administration

Items	Correct	Incorrect	Skipped
	N (%)	N (%)	N (%)
Showed injection sites	160 (94.6)	4 (2.4)	2 (1.2)
Showed injection site rotations	116 (69.9)	46 (27.7)	4 (2.4)
Showed how to shake NPH	92 (55.4)	44 (26.5)	30 (18.1)
Showed how to pinch (fold) skin and inject with (45^o^)	108 (65.1)	54 (32.5)	4 (2.4)
Showed how to draw insulin from the vial	86 (51.8)	49 (29.5)	31 (18.7)

## Discussion

When assessing patients’ knowledge and practice levels, our study revealed that the overall median knowledge of the study samples on insulin storage and handling techniques were moderately adequate, whereas the practice levels were fair. The overall knowledge and practice scores represented as a median of all the knowledge and practice levels by summing up the individual patients’ scores. Proper knowledge and good practices in regards to insulin storage and administration have been an important steps forward to prevent the acute and chronic insulin administration-related complications of DM. Most of DM patients lacked awareness and usually fail to scrutinize the consequences of bad insulin handling practices and poor management skills. The knowledge levels observed in our study was almost comparable with reports (62.13%) from another study conducted in Ethiopia [23], but higher than reports (57.55%) from Nepal [24] and India [19]. However, the practice levels found in our study was lower than what is reported in Nepal (73.98%) [24]. This discrepancy could be partly due to, the patients in the study areas might be less committed, and not confident in their practices. In addition, there might be little reinforcement has been reflected by Health care professionals (HCP) for what patients did. For improving knowledge and practice levels, patients should be compliant to instructions and are still in need of further professional interventions in terms of instructing the recommendations. Previously published studies revealed that DM patients have poor knowledge of disease management and self-care practices [25, 26].

In the present study, residency, payment status to get insulin, mocked demonstrations of injection technique during the first visit, education levels, duration of insulin therapy and duration of illness affected the patients’ knowledge levels. Similar to the study done in Nepal, educated patients (vs not educated) are very likely to understand and practice instructions and commands of storage and administration techniques [24]. Similarly, participants who stayed with the disease longer, and those who took insulin for a longer period of time had better knowledge levels, which enabled them to do the recommended practices. This illustrates that more frequent insulin self-administration might improve the patients’ knowledge and practice levels. It is clear that as time spent on insulin therapy increases, their exposures to information also increases. Moreover, patients might have get an opportunity to learn from the bad consequences of poor insulin handling manners. The findings of the present study were aligned with what Surendranath A. et al. reported, which stated that the patients’ level of knowledge meaningfully influenced the duration of insulin self-administration techniques (*P* < 0.05) [19]. According to studies, numerous recommendations on the insulin injections and storage had no enough logical and scientific supports, but rather it is based on the community habits and traditions [2, 10]. Insulin handling knowledge, practice and skills can be significantly improved if patients are trained with both verbally and practically. This is because applying both verbal and practical trainings could be a complement with each other and help patients to easily understand and remember. Practical mock demonstration of the administration procedures at first visit had positive effects on the respondents’ level of knowledge for insulin storage and administration techniques. In the diabetic self-care process, allowing patients to do mock injection demonstrations in a private diabetic injection training room during their first encounter could possibly build their confidence.

Patients might benefit from regular assessments of their storage and injection practices. In addition, reinforcing them on these practices in subsequent follow-up visits could enhance their knowledge and practice levels than their counterparts. From the occupation perspective, students had the best practice levels than others did. This is possibly, students can read and search the given instructions and recommendations. Besides, they might request clarification on instructions and recommendations that they could not understand. The correlation coefficient test showed a positive linear relationship between knowledge and practice levels of insulin injection. In addition, the Kruskal-Wallis H test also revealed that the patients’ knowledge levels had significant effects on practice levels and handling skills. These all indicate that patients’ injection practice levels may improve with better patients’ knowledge levels in insulin handling techniques.

From the practical skill observational checklist, which assessed the insulin-self administration technique, a significant number of respondents showed the injection sites (94.6%), properly indicated how to rotate the injection sites (70%), and practiced the injection sites rotations (60.8%). This indicates that most of the patients were aware that regular injection site rotations could prevent painful injections, and lipodystrophy, and safeguard the normal tissue for normal absorption. This is supported by a number of articles [10, 27, 28].

Our study revealed that 73.3% of the patients mixed the cloudy insulin (NPH) prior to use. Proper preparation of NPH involves tipping or rolling the vial 20 times. However, we found that only 55.4% of the patients correctly shake the NPH vail. Adequate numbers of rolls or tips of the insulin vails allowed patients to make the suspensions to solutions. Failure to do this could make uneven concentrations of insulin that may possibly lead to hypo- or hyperglycemia. The practices of mixing insulin before use in the present study seem higher than what was reported in India (66.3%). Also, higher numbers of patients (97%) in India [28] tip or roll the vial 10 times or less. The difference of the results between our study and reports from India might be attributed to the smaller sample size of the present study.

Generally, about half of the respondents either performed incorrectly or skipped very important practical skills and critical steps of insulin delivery recommendations. Even though, the study results indicated that the patients’ knowledge and practice level were moderately adequate and fair, respectively, their practical skills were significantly poor. Herein, patients might be unwilling to practice what they had already known and counseled by professionals, or they had forgotten and might have difficulties of in remembering all critical steps. In addition, diabetic patients are commonly frustrated and stigmatized for the needle injections, which could be a driving force for patients to search for other means of insulin delivery rather than injections. This is also might be another challenge for the patients and possibly render them not to be passionate about adapting the appropriate instructions of insulin delivery methods and handling practices. Thus, educating and changing DM patients wrong perceptions, beliefs and attitudes towards storage and administering techniques should be an additional goal of professionals [11].

### Limitation

The present study has not left without limitations. Since we included only patients who visited the randomly selected hospitals during the data collection period, not all potential respondents might be included. Due to the small sample size and the limited number of hospitals, it might be difficult to generalize the findings to the multi-cultural and highly diverse Ethiopian population. In addition, as the study was conducted in the hospitals where healthcare professionals were available all the time, patients might feel under pressure as they may think would be blamed for their poor practices.

## Conclusion

DM patients in the present study had moderately adequate knowledge and fair practices on insulin storage and administration techniques. The patients’ skills on the important and critical steps of administrations were poor. This study highlights the need of regular public health education preferably by healthcare professionals as well as other stakeholders so as to sustainably enhance patients’ knowledge, practice and insulin administration techniques, and ultimately enable patient self-care.

## Supplementary information

**Additional file 1: Supplementary material 1.** Data abstraction format (questionnaire).

## Data Availability

All relevant materials and data supporting the findings of this study are contained within the manuscript and the datasets used and/or analyzed during the current study are available from the corresponding author on reasonable request.

## Abbreviations

CI: Confidence interval
DF: Degree of freedom
DM: Diabetic mellitus
FITTER: Forum for Injection Technique and Therapy: Expert Recommendations
IQR: Interquartile range
NPH: Neutral Protamine Hagedorn
T1DM: Type one diabetic mellitus
T2DM: Type 2 diabetic mellitus
MD: Mean difference
SD: Standard deviation
SPSS: Statistical packages for social science
