# Supplementary material for: Evaluations of knowledge, skills and practices of insulin storage and injection handling techniques of diabetic patients in Ethiopian primary hospitals
Source: BMC Public Health. 2020 Oct 12;20:1537. doi: 10.1186/s12889-020-09622-4 (PMC7552567; doi:10.1186/s12889-020-09622-4)
Supplement: Supplementary file 1 — Additional file 1: Supplementary material 1. Data abstraction format (questionnaire). [file 12889_2020_9622_MOESM1_ESM.docx]

**Data abstraction format**

A data abstraction format prepared to collect data to assess **“Evaluations of** **knowledge, skills and practices of insulin storage and injection handling techniques of patients in Ethiopian primary hospitals**”.

1. Demographic characteristics of patients
2. Age in year ___________
3. Sex: 1. Male 2. Female
4. Residential area: 1. Urban 2. Rural
5. Marital status:
6. Single 2. Married 3. Divorced 4. Widowed/ widower
7. Occupation:
8. Farmer 2. Employer 3. Merchant 4. Housewife 5. Student
9. Educational status:
10. Illiterate 2. Read and write only 3. Primary (grades 1-8) and secondary (grades 9-12) education 4. College and above
11. How do you get your insulin? 1. For free 2. Payment
12. For how long have you been with diabetes (in year) ___________
13. For how long have you been on insulin therapy (in year) ___________
14. Do healthcare professionals trained on how to administer insulin? 1. Yes 2. No
15. Were you trained during your first insulin prescription? 1. Yes 2. No
16. Did you demonstrate for the healthcare professionals how was the insulin administration technique has been performed during your first training? 1. Yes 2. No
17. How were you instructed on how to administer insulin?
18. Only oral instructions 2. Only practical demonstrations 3. Both ways
19. Table 1: Items used to assess Insulin handling and injection experiences and practices of participants

| No. Items | Never | Some  times | Often/  usually | Always |
| --- | --- | --- | --- | --- |
| 1. How many times you checked the expiry date of the insulin vial or cartridge? |  |  |  |  |
| 1. How many times you avoided the extremes of cold or heat temperatures of insulin storage? |  |  |  |  |
| 1. How many times were you mixed well cloudy (NPH) insulin prior to use? |  |  |  |  |
| 1. How many times you discarded if the insulin is discolored? |  |  |  |  |
| 1. How many times do you use clumpy/frosted insulin after it has been thawed? |  |  |  |  |
| 1. How many times you inspected insulin before inject it? |  |  |  |  |
| 1. How many times you labeled a newly opened vial and indicating the date of discarding: |  |  |  |  |
| 1. How many times you used the opened (starting from stick a needle in the vial) insulin after 28 days? |  |  |  |  |
| 1. How many times you removed insulin kept in the fridge and allowed to reach room temperature before injection? |  |  |  |  |
| 1. How many times you injected insulin through your clothing? |  |  |  |  |
| 1. How many times you wiped off the top of the bottle with alcohol Swab |  |  |  |  |
| 1. How many times you washed your hands and injection site before injection? |  |  |  |  |
| 1. How many times you cleaned the injection site prior to injection? |  |  |  |  |
| 1. How many times you rotated the injection site? |  |  |  |  |

Table 2: Items used to assess Knowledge of insulin storage and administration techniques of participants

| No. Items | Yes | No |
| --- | --- | --- |
| 1. Do you keep the vials and cartridges of insulin not currently (unopened) use in the refrigerator (2 to 8 degrees) or (traditional equivalent methods) until their date of expiry away from freezing coils or freezer? |  |  |
| 1. Do you store the vials and cartridges, which are in current use (opened), at cool and dark places of room temperature (15-25ºC) or traditional equivalent methods? |  |  |
| 1. Do you discard unopened insulin after 28 days of use? |  |  |
| 1. Do you keep currently being used cartridges in the refrigerator? |  |  |
| 1. Do you gently roll the vial between the palms of the hands and /or moving the insulin up and down 20 times? |  |  |
| 1. Is shaking or jarring of insulin containers can make insulin more likely to frost or clump? |  |  |
| 1. Do you squirt back anexcess amount of insulin into the vial? |  |  |
| 1. Do you keep insulin vials or cartridges in the glove box of a car? |  |  |
| 1. Is the out dated (expired) insulin recommended to be ever used? |  |  |
| 1. Could exposing the insulin containers to a high temperatures/light/heat alter the effectiveness of insulin? |  |  |
| 1. Is the cold insulin may sting and the action could be delayed? |  |  |
| 1. Do you keep insulin in freeze states of the temperature? |  |  |
| 1. Are the injection sites rotation distances on the same site measured with the thump? |  |  |
| 1. Are the recommended insulin injection measures used to avoid or minimize pain caused by injections? |  |  |

Table 4: Observational checklist of patients’ skill related to self-insulin administration

| No. Items | Correct | Incorrect | Skipped |
| --- | --- | --- | --- |
| 1. Showed injection sites |  |  |  |
| 1. Showed injection site rotations |  |  |  |
| 1. Showed how to shake NPH |  |  |  |
| 1. Showed how to pinch (fold) skin and inject with (45^o^) |  |  |  |
| 1. Showed how to draw insulin from the vial |  |  |  |
